# Efficacy of adding ramipril (VAsotop) to the combination of furosemide (Lasix) and pimobendan (VEtmedin) in dogs with mitral valve degeneration: The VALVE trial

**DOI:** 10.1111/jvim.15863

**Published:** 2020-09-18

**Authors:** Gerhard Wess, Jan‐Gerd Kresken, Ralph Wendt, Juliane Gaugele, Markus Killich, Lisa Keller, Julia Simak, Peter Holler, Alexander Bauer, Helmut Küchenhof, Tony Glaus

**Affiliations:** ^1^ Clinic of Small Animal Medicine Ludwig‐Maximilians‐Universität Munich Germany; ^2^ Clinic for Small Animals Kaiserberg Duisburg Germany; ^3^ Tierärztliche Überweisungspraxis Kirschenwäldchen Wetzlar Germany; ^4^ Statistical Consulting Unit, StaBLab, Department of Statistics Ludwig‐Maximilians‐Universität Munich Germany; ^5^ Division of Cardiology, Department of Small Animals, Vetsuisse Faculty University of Zurich Zurich Switzerland

**Keywords:** ACEI, CHF, degenerative mitra valve disease, mitral regurgitation, therapy

## Abstract

**Background:**

Triple therapy (TT) consisting of furosemide, pimobendan, and an angiotensin‐converting enzyme inhibitor (ACEI) frequently is recommended for the treatment of congestive heart failure (CHF) attributable to myxomatous mitral valve disease (MMVD). However, the effect of adding an ACEI to the combination of pimobendan and furosemide (dual therapy [DT]) so far has not been evaluated prospectively.

**Hypothesis:**

Triple therapy will extend survival time compared to DT in dogs with CHF secondary to MMVD.

**Animals:**

Client‐owned dogs presented with the first episode of CHF caused by MMVD.

**Methods:**

Prospective, single‐blinded, randomized multicenter study. One‐hundred and fifty‐eight dogs were recruited and prospectively randomized to receive either DT (furosemide and pimobendan) or TT (furosemide, pimobendan, and ramipril). The primary endpoint was a composite of cardiac death, euthanasia for heart failure, or treatment failure.

**Results:**

Seventy‐seven dogs were randomized to receive DT and 79 to receive TT. Two dogs were excluded from analysis. The primary endpoint was reached by 136 dogs (87%; 66 dogs, DT; 70 dogs, TT). Median time to reach the primary endpoint for all dogs in the study was 214 days (95% confidence interval [CI], 168‐259 days). Median time to reach the primary endpoint was not significantly different between the DT group (227 days; interquartile range [IQR], 103‐636 days) compared with TT group (186 days; IQR, 72‐453 days; *P* = .42).

**Conclusions and Clinical Importance:**

Addition of the ACEI ramipril to pimobendan and furosemide did not have any beneficial effect on survival time in dogs with CHF secondary to MMVD.

**Abbreviations**
ACEI: angiotensin‐converting enzyme inhibitor
Ao: aortic root diameter
ARB: angiotensin receptor blocker
BW: body weight
CHF: congestive heart failure
CKCS: cavalier King Charles spaniels
DCM: dilated cardiomyopathy
DT: dual therapy
FS: fractional shortening
HFpEF: patients with preserved ejection fraction
HFrEF: heart failure with reduced ejection fraction
LA: left atrium
LA/Ao: left atrial‐to‐aortic root ratio
LVIDD: left ventricular internal end‐diastolic dimension
LVIDDN: left ventricular internal diameter at end diastole normalized to bodyweight (kg)
LVIDS: left ventricular internal end‐systolic dimension
LVIDSN: left ventricular internal diameter at end systole normalized to bodyweight (kg)
MMVD: myxomatous mitral valve disease
PE: pulmonary edema
TT: triple therapy
VHS: vertebral heart score

## 1 INTRODUCTION

Myxomatous mitral valve degeneration is the most common cardiac disease in small dogs, and prevalence increases with age.^1^, ^2^ In the course of the disease, dogs eventually develop clinical signs of congestive heart failure (CHF).^3^ After having suffered from an episode of CHF, most dogs die within 1 year despite medical management.^4^, ^5^, ^6^ Although there has been much research interest in myxomatous mitral valve disease (MMVD) and CHF, it is still unclear which treatment protocol is optimal. Although it is clear that the combination of pimobendan and furosemide is superior to the combination of an angiotensin‐converting enzyme inhibitor (ACEI) and furosemide, it is unknown if the addition of an ACEI to pimobendan and furosemide provides additional benefit to pimobendan and furosemide alone. In the QUEST study, dogs with MMVD and CHF were randomized to receive either pimobendan or benazepril in addition to diuretic treatment. Median time to endpoint was significantly longer in the pimobendan group (267 days) than in the benazepril group (140 days).^6^ These results show that dual therapy (DT), consisting of pimobendan plus furosemide, is superior to an ACEI (benazepril) together with a diuretic in dogs with MMVD and CHF. Short‐term studies evaluating ACEI in dogs with MMVD and dilated cardiomyopathy (DCM) showed an improved quality of life.^7^, ^8^ Subsequently, the LIVE and BENCH study groups confirmed prolonged survival times for dogs receiving an ACEI in addition to diuretics compared with placebo.^4^, ^5^ Therefore, considering the potential positive effects of an ACEI on the pathophysiological processes in CHF, triple therapy (TT) (ie, a treatment regime combining diuretics with pimobendan and an ACEI) might be superior to DT. Likewise, the 2009 ACVIM guidelines for the diagnosis and treatment of chronic valvular heart disease in dogs recommended TT consisting of furosemide, pimobendan, and an ACEI as a consensus recommendation for the chronic management of CHF.^9^ In the current ACVIM guidelines, TT is still recommended,^10^ but no studies have prospectively investigated the advantages of TT compared to DT with regard to mortality and prognosis. Therefore, our aim was to evaluate if a positive effect could be achieved by adding the ACEI ramipril to DT with respect to mortality and prognosis in dogs with MMVD and CHF.

## 2 MATERIAL AND METHODS

### 2.1 Animals

Dogs experiencing their first episode of CHF caused by MMVD were prospectively recruited at 4 centers in Europe by investigators specialized in veterinary cardiology (board‐certified American College of Veterinary Internal Medicine [ACVIM] or European College of Veterinary Internal Medicine [ECVIM] or German national cardiology specialization title, Collegium Cardiologicum). All dogs were client‐owned and were enrolled into the study between 2005 and 2015. The study was concluded in 2016.

## 3 ENROLMENT CRITERIA

#### 3.1.1. Inclusion criteria

To be eligible, owners had to give written consent to participate in the study. Thoracic radiographs were used to confirm evidence of cardiogenic pulmonary edema (PE), based on a perihilar, diffuse or focal interstitial or alveolar lung pattern,^11^ and advanced MMVD based on cardiomegaly with a vertebral heart score (VHS) > 10.5, as well as a moderately to severely enlarged left atrium. Clinical signs of decompensated CHF such as tachypnea (respiratory rate > 50/min in the examination room or resting respiratory rate > 40/min at home, or both) and dyspnea had to be present or must have resolved in the past with diuretic treatment (while still being administered and considered necessary to maintain a compensated state by the attending clinician). The first CHF episode must not have occurred earlier than 30 days before inclusion in the study. All dogs had to have a characteristic moderate to high intensity systolic heart murmur with maximal intensity over the mitral area. The diagnosis of advanced MMVD additionally had to be confirmed using echocardiography, based on typical qualitative findings in 2‐dimensional (2D) echocardiography (ie, thickened mitral valve leaflets, with or without prolapse or ruptured chordae tendineae), color Doppler (marked mitral regurgitation), and quantitative left atrial and left ventricular enlargement (ie, LA/Ao > 1.5, left ventricular internal dimension in diastole [LVIDd] above the normal reference range).^2^, ^12^, ^13^ All dogs had to be ≧5 old and had to have a body weight (BW) between 5 and 20 kg to ensure appropriate drug dosing.

#### 3.1.2. Exclusion criteria

Dogs with either a history of clinically relevant systemic disorders (eg, liver, gastrointestinal, or renal disease not consistent with prerenal azotemia) or a congenital or acquired heart disease other than MMVD were excluded. Dogs with tricuspid valvular insufficiency attributable to myxomatous valve disease were allowed to be included if present together with clinically relevant mitral regurgitation in which the latter was judged to be the major contributor to the presenting signs of disease. Pregnant or lactating female dogs were excluded.

#### 3.1.3. Study design

The “Comparative investigation into an Extension of Survival Time Benefits of ramipril (**V**asotop) in **a**ddition to furosemide (**L**asix) and pimobendan (**V**etmedin) in canine **E**ndocardiosis” (VALVE Trial) study design was similar to the QUEST trial study design^6^ and initiated by the principal investigator (Gerhard Wess) as a single center study. After receiving funding 3 years after the start of the study, 3 additional study centers were recruited as additional investigators. The study was conducted according to Good Clinical Practice. The investigators had full access to all results and performed the statistics independently from the sponsor.

#### 3.1.4. Randomization and allocation

The trial was designed as a prospective, randomized, positive‐controlled, single‐blinded, multicenter study. Block randomization was used with a 1 : 1 allocation ratio to maintain similar sample sizes in both treatment groups. The study numbers were grouped into blocks of 10, and each study number within a block was randomly assigned to a treatment group (DT or TT) by computer software.

#### 3.1.5. Blinding

To keep the investigators blinded, a separate dispenser handed the study treatment to the owner. Each dispenser selected the treatment according to the BW and randomization list. The owner was not allowed to talk about the trial treatments with anyone except the dispenser. The investigators, study monitors and the sponsor remained blinded for the duration of the study. Unblinding only occurred after completion of the study and data entry.

#### 3.1.6. Trial treatments

The dogs were randomly allocated in treatment groups to receive TT (furosemide, pimobendan [Vetmedin, 1.25 mg, 2.5 mg Boehringer Ingelheim Vetmedica GmbH, Ingelheim/Rhein, Germany] plus ramipril [Vasotop 0.625 mg, Vasotop 1.25 mg, Vasotop 2.5 mg, and Vasotop 5 mg, Intervet Deutschland GmbH, Unterschleißheim, Germany]) or DT (furosemide and pimobendan). The study treatment was given according to the manufacturer's recommendations (pimobendan, 0.2‐0.3 mg/kg PO q12h; Ramipril, 0.125‐0.25 mg/kg PO q24h) by adjusting the dose to a suitable number of capsules or tablets. The administration frequency for ramipril could be doubled if judged clinically necessary and advised by the investigator. To ensure that the investigator remained blinded to treatment allocation of the case, the investigator instructed the dispenser in these cases, “If the dog is receiving ramipril, please double the dose.”

#### 3.1.7. Concomitant treatments

All medications received at the time of enrollment or administered during the course of the study were documented. If a dog already was receiving an ACEI, the drug was discontinued if the dog was randomized to the DT group or switched to ramipril if the dog was allocated to the TT group and had been receiving a different ACEI.

Regarding concomitant therapy, all drugs were allowed except calcium channel antagonists (eg, diltiazem, verapamil, amlodipine), xanthines (eg, theophylline derivatives, etamiphylline, propylxanthin/propentofylline), angiotensin II receptor blockers, or other ACEIs.

#### 3.1.8. Schedule of events

The medical history of each dog was established with the owner and results of available thoracic radiographs were reviewed by each investigator for the presence of PE and a moderately to severely enlarged left atrium, if the first episode of CHF (PE) had been diagnosed by a referring veterinarian. All dogs were subjected to complete physical examination, thoracic radiographs, electrocardiography, echocardiography, and selective blood analyses (serum creatinine, potassium [K+], sodium [Na+], and total protein [TP] concentrations). Reexaminations were scheduled at day 7 (±1 day), day 28 (±3 days), and 3 months (±1 week) after initial examination. Thereafter, the dogs were reexamined every 3 months (±2 weeks) until death, euthanasia, or withdrawal from the trial. The date of death or withdrawal was recorded.

#### 3.1.9. Quality of life and respiratory variables

Quality of life variables (eg, appetite, demeanor, activity level) and respiratory variables (eg, respiratory effort, coughing) were evaluated at the first presentation and scored according to the system presented in Table 1.

**TABLE 1:** Quality of life and respiratory variables

Variable	Score	Clinical correlation
**Appetite**	1	Increased
	2	Normal
	3	Decreased (about 2/3 of normal)
	4	Markedly decreased (less than 2/3 of normal)
**Demeanor**	1	Alert, responsive
	2	Mildly lethargic
	3	Moderately lethargic
	4	Minimally responsive
	5	Unresponsive
**Activity level**	1 (Very good)	Dog moves around with ease, capable of climbing stairs, alert and responsive to external stimuli, dog is able to do normal exercise
	2 (Good)	Dog moves around with ease, capable of climbing stairs, alert and responsive to external stimuli, dog is not able to do full exercise, ability of running is reduced
	3 (Moderate)	Dog is less active than normal, moves around a few times per day, has difficulties with stairs, avoids long walks
	4 (Poor)	Dog is inactive, only gets up to eat, drink or urinate, is unable to climb stairs
**Respiratory effort**	1	Normal
	2	Mildly increased respiratory rate or effort
	3	Moderately labored
	4	Severe respiratory distress
**Cough**	1	None
	2	Occasional (a few times a week)
	3	Frequent (a few times each day)
	4	Persistent (frequently during the day)
**Pulmonary edema score**	1	None
	2	Mild interstitial opacity
	3	Moderate interstitial opacity
	4	Alveolar pattern
	5	Severe consolidation

### 3.2 Circulatory variables

#### 3.2.1 Heart rate and ECG

A 3‐minute 6‐lead ECG was recorded with the dog in right lateral recumbency and heart rate was reported in beats per minute. The findings of the ECG were categorized as no abnormal findings (ie, sinus rhythm), arrhythmia, atrial fibrillation, or other findings.

#### 3.2.2 Echocardiography

Echocardiography was performed on unsedated dogs in right and left lateral recumbency with a simultaneous ECG tracing according to the recommendations of the Echocardiography Committee of the Specialty of Cardiology, ACVIM.^14^ Each variable was measured at least 3 times and mean values were used for further analysis.^14^ From the 2D image, LA/Ao was measured at the level of the heart base in the right parasternal short axis view as previously described.^12^ The M‐mode measurements (left ventricular internal end‐diastolic dimension [LVIDD], left ventricular internal end‐systolic dimension [LVIDS], fractional shortening [FS%]) were obtained in the right parasternal short axis view, using 3 consecutive cardiac cycles.^13^ The LVIDD was normalized to BW (LVIDDN) using the formula: LVIDDN = LVIDD (cm)/weight^0.294^ (kg) and LVIDS using the formula: using the formula: LVIDSN = LVIDS (cm)/weight^0.315^ (kg).^13^

#### 3.2.3 Thoracic radiography

Thoracic radiography was performed at presentation using right lateral and ventrodorsal projections or radiographs from referring veterinarians were reviewed to confirm if PE had been present previously, in order to be eligible to enter the study. The VHS method was used to quantitate cardiac size,^15^ and the presence and severity of PE (none, mild interstitial density, moderate interstitial density, alveolar pattern, and severe consolidation) were assessed.

#### 3.2.4 Withdrawal

Reasons for removing an animal from the study were noncompliance of the owner causing study procedure complications, adverse reactions that led to discontinuation of study drugs, or illness or injury of the patient that interfered with the study.

#### 3.2.5 Endpoint

The outcome of the dogs was evaluated by follow‐up examinations or telephone interviews with the owner. The primary endpoints of the trial were sudden cardiac death, euthanasia for CHF, or treatment failure. When a dog died spontaneously or was euthanized, the investigator specified whether the cause of death was considered to be cardiac or noncardiac. In cases in which the cause of death was considered noncardiac, the reason for death or euthanasia was noted. Treatment failure was assumed if a dog showed the following clinical signs despite receiving the maximum allowed study dosage of furosemide (15 mg/[kg d]) and spironolactone (4 mg/[kg d]): persistent dyspnea, progressive ascites, severe cardiac cachexia, or severe exercise intolerance. Survival time was defined as the time period from enrollment into the trial to an endpoint.

### 3.3 Statistical analysis

Power calculations were based on data from the QUEST study.^6^ It was assumed that TT would increase median survival time to 1 year compared to approximately 9 months under DT. Estimating an event rate at 1 year of 75% with DT as opposed to 50% with TT, with a power of 80%, an α of .05 and a β of .2, the minimal number of patients was calculated at 58 dogs per group. To compensate for possible dropouts, a sample size of 75 dogs was chosen for each group.

A Wilcoxon's signed‐rank test was used to compare the continuous baseline variables at enrollment to assess for significant differences between treatment groups. For categorical data, a Fisher's exact test was used.

To determine whether a significant difference existed between the 2 treatment groups, a log‐rank test with right censoring was used, and the Kaplan‐Meier method was applied to estimate the median time to endpoint for each treatment group and plot time‐to‐event curves. For each baseline variable, univariate Cox proportional hazards analysis with right‐censoring was performed to determine whether any baseline variable was associated with time to endpoint and the hazard ratio (HR) and 95% confidence intervals (CI) were calculated.

Multivariate Cox proportional hazard analyses were performed in a backward stepwise manner. The analyses started with treatment group and then all other baseline variables from the univariate analysis were included in the model. The variable with the highest *P* value was eliminated at each step, with reanalysis between steps, until the final model was obtained. All variables were assessed only as main effects; no interaction terms were considered in modeling.

Additionally, a Cox‐model with least absolute shrinkage and selection operator (LASSO) penalization was performed.^16^, ^17^ Penalization means that variables with a very small effect are “punished” and set to zero, so that only relevant variables remain for a normal Cox model analysis.

For all analyses, a *P* value ≤.05 was considered significant. All analyses were 2‐tailed. Median values and interquartile ranges (IQR) are reported. Statistical analyses were performed using the open‐source software R and the commercially available software PASW Statistics (R (Version 3.1.2) (R Core Team [2014]. A language and environment for statistical computing. R Foundation for Statistical Computing, Vienna, Austria. URL http://www.R-project.org/; PASW Statistics, Version 18.3.1, IBM Corporation, Armonk, New York). Cox models were estimated using the function “coxph” from the R package “survival”^18^; the LASSO based Cox model was estimated with the function “glmnet” from the R package “glmnet”^16^ using commercially available software programs (R Foundation for Statistical Computing, Vienna, Austria. URL http://www.R-project.org/; PASW Statistics, Version 18.3.1, IBM Corporation).

## 4 RESULTS

### 4.1 Baseline data

One‐hundred and fifty‐eight dogs were recruited. Two dogs were excluded from further analysis after the termination of the trial; 1 dog because of violation of inclusion criteria (BW of 3.2 kg) and 1 owner failed to adhere to the schedule for reexaminations by >90 days. Of the remaining 156 dogs, 93 were males and 63 females. The most common recruited breeds were Dachshunds (n = 37), Poodles (n = 11), Jack Russell Terriers (n = 9), cavalier King Charles spaniels (CKCS; n = 8), Yorkshire Terriers (n = 6), and Chihuahuas (n = 6). Thirty‐one other breeds with 1 to 5 dogs each also were represented and there were 41 mixed breed dogs. Of the 156 dogs, 77 received DT and 79 TT. The baseline characteristics at enrollment are shown in Table 2. The only significant difference between the 2 treatment groups was that 20 dogs (26%) in the DT vs 35 dogs (44.3%) in the TT group had received an ACEI before enrollment. The median dose of pimobendan was 0.5 mg/(kg d) (IQR, 0.46‐0.58) and the median dose of ramipril was 0.21 mg/(kg d) (IQR, 0.16‐0.23) at entry into the study. Ramipril was doubled upon the discretion of the clinician in 3 dogs during the study.

**TABLE 2:** Summary of baseline characteristics in the 2 treatment groups

	Variable	Dual therapy (n = 77)	Triple therapy (n = 79)	*P* value
Dog characteristics	Age (y)	11 [9‐12]	11 [9.8‐12.3]	.54
	Sex (female/male) (%)	25/52 (32.5%/67.5%)	38/41 (48.1%/51.9%)	.06
	Cavalier (no/yes) (%)	73/4 (94.8%/5.2%)	76/3 (96.2%/3.8%)	.72
Duration of clinical signs	CHF (d)	7 [1.25‐15]	3 [0.5‐11]	.53
Pretrial treatment	ACEI, no/yes (%)	57/20 (74.0%/26,0%)	44/35 (55.7%/44.3%)	.02
	Time pretrial ACEI (mo.)	9.0 [4.0‐16.0]	9.0 [3.0‐24.0]	.83
Treatment day 1 (in addition to group treatment)	Furosemide dose (mg/[kg d])	8.2 [4‐10.8]	8.0 [4.9‐11.2]	.89
	Digoxin (number of dogs)	2	3	.94
	Spironolactone (number of dogs)	3	2	.96
Quality of life and respiratory variables	Appetite	2 [2‐3]	2 [2‐3]	.44
	Demeanor	2 [2‐3]	2 [1‐3]	.28
	Respiratory effort	3 [2‐4]	3 [2‐3]	.67
	Cough	3 [2‐3]	3 [2‐3]	.45
	Exercise tolerance	3 [3‐3.5]	3 [2‐3]	.41
Physical examination	Weight (kg)	9.0 [7.2‐11.8]	8.3 [6.3‐10.4]	.14
	Heart rate (bpm)	152 [140‐162.5]	150 [130‐160]	.47
	Rectal temperature (°C)	38.4 [38.1‐38.6]	38.5 [38.2‐38.7]	.68
Laboratory variables	Sodium (mM/L)	148 [144‐150]	147 [144‐150]	.85
	Potassium (mM/L)	4.5 [4‐5.1]	4.5 [4.1‐5]	.99
	Creatinine (μM/L)	58 [5‐81]	63 [47‐87]	.35
	PCV (%)	50 [45‐53]	49 [45‐53]	.74
	TPC (g/L)	58 [7.1‐67]	63 [50‐70]	.13
Diagnostic Imaging	VHS	12 [11.1‐12.5]	12 [11.5‐12.6]	.08
	PE (no/yes)	2/75	0/77	.79
	PE score (1‐5)	4 [2‐4]	4 [2‐4]	.57
	LVIDSN (cm/kg^0.315^)	1.11 [0.99‐1.24]	1.11 [0.96‐1.28]	.98
	LVIDDN (cm/kg^0.294^)	2.08 [1.89‐2.28]	2.07 [1.89‐2.25]	.79
	FS (%)	43.6 [40.0‐48.0]	44 [38.7‐50.6]	.68
	LA/Ao	2.3 [2.0‐2.6]	2.3 [2.0‐2.7]	.57

*Note*: Data are indicated as medians [and interquartile range] or absolute numbers (and frequencies in %).

Abbreviations: ACEI, angiotensin‐converting enzyme inhibitor; bpm, beats per minute; CHF, congestive heart failure; FS, fractional shortening; HF, heart failure; LA/Ao, left atrial to aortic root ratio; LVIDDN, left ventricular internal dimension in diastole normalized; LVIDSN, left ventricular internal dimension in systole normalized; PCV, packed cell volume; PE, pulmonary edema; TPC, total protein concentration; VHS, vertebral heart score.

### 4.2 Overall outcome

Of the 156 included dogs, 136 (87.2%) dogs reached the primary endpoint: 82 (52.6%) were euthanized because of refractory heart failure, 47 (30.1%) died spontaneously of cardiac causes, and 7 (4.5%) reached the treatment failure endpoint. The event rate therefore was 87%. The median time to reach the primary endpoint for all dogs in the study was 214 days (CI, 168‐259 days).

Twenty dogs (12.8%) were censored: 10 (6.4%) dogs were alive at the termination of the trial (5 dogs each in DT and TT group), 5 (3.2%) dogs were euthanized for noncardiac reasons (4 dogs for neoplasia; 2 in the DT and 2 in the TT group; 1 dog for neurologic signs in the DT group), and 5 (3.2%) were censored because of owner noncompliance (2 dogs in the DT and 3 in the TT group).

### 4.3 Effect of treatment on outcome

No significant differences were found with respect to the proportions of dogs reaching the different endpoints between the DT and TT groups (*P* = .83). The primary endpoint euthanasia for cardiac reasons was similar between the treatment groups: 42/77 (54.5%) dogs in the DT group and 40/79 (56.6%) in the TT group. Cardiac death occurred in 24/77 (31.2%) and 23/79 (29.1%) dogs in the DT and TT cohorts, respectively. Treatment failure occurred in 6 dogs in the TT group and in 1 dog in the DT group (Table 3). Spironolactone was prescribed as an additional drug after entry into the study in 13 dogs, 5 in the DT and 8 in the TT group.

**TABLE 3:** Comparison between treatment groups (censored dogs excluded) for the median time (interquartile range) to reach the endpoint for each of the individual endpoints, which were combined to create the composite primary endpoint of the study

Endpoints	Median time to endpoint (d)		Log‐rank *P* value
			Fisher's exact test *P* value
	Dual therapy	Triple therapy	
All endpoints	214 (101‐456) n = 67	148 (63‐342) n = 69	.39 .74
Euthanasia for cardiac reason	206 (101‐486) n = 42	189 (56‐356) n = 40	.87 .12
Cardiac death	228 (96‐390) n = 24	125 (62‐344) n = 23	.34 .89
Treatment failure	116 (116‐116) n = 1	78 (75‐149) n = 6	.76 .09

*Note*: The log‐rank test was used to compare the survival times and Fisher's exact test to compare the percentage of dogs reaching each endpoint.

**FIGURE 1:**
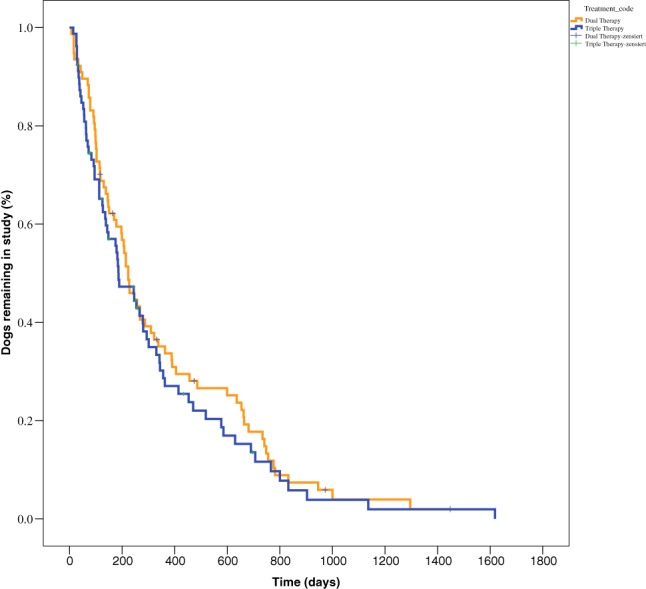
Kaplan‐Meier plot of percentage of dogs in the study as a function of time in 77 dogs treated with dual therapy (DT) and in 79 dogs treated with triple therapy (TT). The median time to reach the primary endpoint was not significantly different between the DT group (227 days; IQR, 103‐636 days) and the TT group (186 days; IQR, 72‐453 days) (*P* = .42). IQR, interquartile range

The median time to reach the primary endpoint was not significantly different between the DT group (227 days; IQR, 103‐636 days) compared with TT group (186 days; IQR, 72‐453 days; *P* = .42; Figure 1). Ten dogs each were censored in the DT group (13%) and the TT group (12.7%), which was not significantly different (*P* = .87).

### 4.4 Univariate and multivariate cox proportional hazard analyses

**FIGURE 2:**
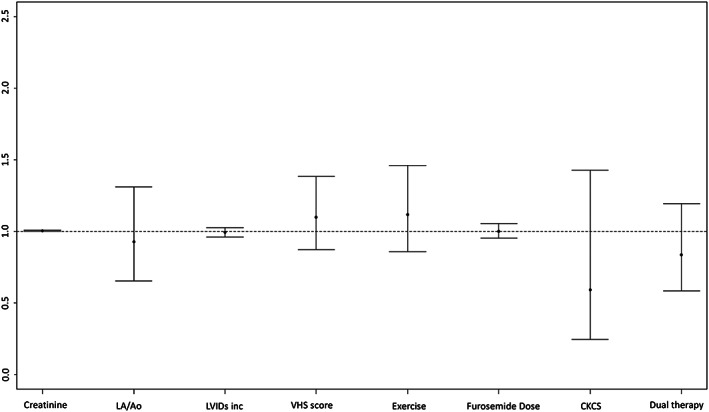
Hazard ratios from the multivariate cox proportional hazard analyses showing the effect of various variables at baseline and treatment on survival

The effects of treatment and baseline variables were tested in a univariate and multivariate analysis. Neither treatment group nor any other variable had a significant effect on outcome (Figure 2). The only significant different variable between the DT and TT groups at inclusion, pretrial treatment with ACEI, had no effect on outcome (HR = 1; *P* = .98).

To evaluate if any of the 3 composite endpoints had an effect on outcome, a subgroup analysis was performed for the noncensored dogs (Table 3). For each of these subgroups, no significant difference in median time to endpoint was found for the DT dogs compared with the TT dogs.

## 5 DISCUSSION

The use of ACEI in addition to pimobendan and a diuretic for treatment of CHF has been and still is recommended based on expert opinion and pathophysiological assumptions.^9^, ^10^ Our study shows, however, that addition of an ACEI to DT did not prolong median time to cardiac death, euthanasia for cardiac reasons, or treatment failure in dogs with PE caused by MMVD. In fact, median time to the primary endpoint was more than a month longer in the DT group, although this difference was not statistically significant. The results of the DT group in our study (227 days until endpoint) are comparable to the same treatment group in the QUEST trial (267 days until endpoint).^6^ The design of the present VALVE study followed the QUEST study design and therefore the similarity between the DT and the pimobendan group in the QUEST trial is not surprising. Our study had a very high event rate of 87% which is so far the highest event rate reported in a prospective randomized study of MMVD in dogs. The high event rate is explained by the long follow‐up time that allowed so many patients to reach the primary endpoint. This high event rate makes our results highly credible.

Before pimobendan was introduced in veterinary medicine, the use of ACEI in heart failure in dogs had been tested in multiple trials. Two short‐term trials, the IMPROVE and the COVE studies, had included dogs with DCM and MMVD, and both had shown more pronounced beneficial short‐term effects in DCM compared to MMVD concerning heart failure class and PE scores.^7^, ^8^ Two long‐term studies (the LIVE and the BENCH trials) evaluated the effect of ACEIs in dogs with CHF caused by MMVD or DCM. The LIVE study included 67 dogs with MMVD and showed a significantly (*P* = .006) improved mean survival time of 160 days (ACEI plus furosemide) vs 87 days in the furosemide alone group.^4^ The BENCH study included 125 dogs with MMVD; the dogs receiving an ACEI had a significantly longer mean survival time compared with the placebo group (436 vs 151 days, *P* = .01), but in this study not all dogs received diuretics and therefore probably not all dogs were in CHF.^5^ This difference also explains the much longer survival time of the ACEI group in the BENCH trial (436 days) compared with the ACEI group in the LIVE (160 days) and QUEST (140 days) trials.^5^, ^6^

In human medicine, guidelines for the management of patients with valvular heart disease recommend surgery (ie, valve repair or replacement). In contrast, only sparse data exist for medical treatment such as the use of beta‐blockers, ACEI, or angiotensin receptor blockers (ARBs).^19^ The recent American Guidelines for the Management of Heart Failure recommended ACEI for patients in heart failure with decreased ejection fraction (HFrEF) to decrease morbidity and mortality.^20^ In patients with preserved ejection fraction (HFpEF), however, the use of ACEI, beta‐blocking agents, and ARBs is only recommended for patients with systemic hypertension to control blood pressure.^20^ Similarly, the European guidelines recommend an ACEI in addition to a beta‐blocker for symptomatic patients with HFrEF to decrease the risk of heart failure hospitalization and death.^21^ However, the guidelines also state that trials of ACEIs, ARBs, beta‐blockers, and ARBs all have failed to decrease mortality in patients with HFpEF.^21^ Recently, a review examined all mega trials (those involving >1000 patients) and smaller pivotal trials involving ACEI in humans and came to the conclusion that doubts remain over the concept of blood pressure‐independent cardiovascular protection offered by ACEI or ARBs.^22^ Because systemic hypertension is a major health risk factor and comorbidity in humans, but not recognized as such in dogs with MMVD, this might explain why in our study no effect of the ACEI used was identified when added to pimobendan. Potentially beneficial vasodilatory effects of the ACEI used could have been masked in our study by the additional vasodilatory action of pimobendan providing a possible explanation for no significant differences regarding survival times between treatment groups and making the addition of an ACEI to pimobendan treatment nonessential in dogs with MMVD.

Ramipril was chosen as the ACEI in our study, because it was a commonly used product in Europe. One study that compared 5 ACEI (including ramipril) had not found any pharmacokinetic advantage of 1 ACEI over any other. Furthermore, ramipril had been shown to normalize plasma and left ventricular angiotensin II concentrations in dogs with experimental mitral regurgitation.^23^, ^24^

Appropriate dosing is a common concern with ACEI use in dogs. In our study, the median dosage at study entry actually was 1.7 higher than the recommendation of the manufacturer. Therefore, it is unlikely that underdosing of the ACEI is responsible for the results of our study.

The only baseline variable that was different at inclusion between the 2 treatment groups was the period of pretreatment use of ACEI, which was higher in the TT group. The multivariate Cox proportional hazards analysis enables determination of whether or not there is an effect of treatment after accounting (adjusting) for the effect of this difference, and this variable was not a significant contributor to survival time. In contrast to the QUEST study, no variable was significantly associated with increased risk of reaching the composite endpoint in our study. Specifically, in the QUEST study, CKCS, receiving a lower dose of furosemide and having a higher serum creatinine concentration, were associated with a decreased risk of reaching the composite endpoint. However, the number of CKCS was low in our study with only 4 CKCS in each group (ie, 5% of all dogs), whereas in the QUEST study CKCS represented 27% of dogs in the pimobendan and 38% of the dogs in the ACEI group. Unlike in the QUEST trial, in our study, none of the imaging variables were significantly associated with outcome. This finding may be surprising because a larger heart is expected to represent more advanced disease in MMVD and therefore to be associated with shorter survival. On the other hand, it can be argued that the inclusion criterion was the first episode of PE because of MMVD, which implies that the disease was similarly advanced in the individual dogs. Our inclusion criteria followed those of the QUEST study, and therefore the LA/Ao ratio had to be >1.5. However, this cutoff would not likely prove a moderate or marked degree of mitral regurgitation typical of advanced MMVD. Likewise, the lower IQR of the LA/Ao ratio in our study was 2.0, consistent with advanced disease.

## 6 LIMITATIONS

Although our study is 1 of the larger, prospective, and randomized trials in dogs, it still is small when compared to trials in human medicine. Another limitation is the single‐blinded study design, where the owner was not blinded. However, blinding of the investigator was ascertained by using a drug dispenser to interact with the owner concerning all issues relative to the administration of the drugs given in our study, and therefore investigator bias at data acquisition is not considered a concern.

Blood pressure measurement was not a mandatory part of the study protocol, but as it affected both groups and dogs were allocated to groups randomly, high or low blood pressures would have expected to affect both groups. Patients with concurrent systemic diseases potentially predisposing to systemic hypertension or causing marked systemic hypotension were excluded in the first place.

Besides increasing the furosemide dose and adding spironolactone in both treatment groups at the investigators' discretion, treatment modification was only allowed for ramipril, but not in the DT group. This may have introduced bias in favor of the TT group. Also, withdrawal of ACEI from dogs in the DT that had received an ACEI before entry into the study, or the switching of the prescribed ACEI to ramipril in the TT group, might be a limitation. The withdrawal of a long‐term prescribed drug might have a detrimental effect for the patient,^25^ but in our study, this primarily would have affected the DT group suffering potentially from drug withdrawal, whereas the switching to another ACEI product is most likely less problematic. However, despite this theoretical advantage in the TT, it was not superior to DT.

In conclusion, the addition of an ACEI to pimobendan and a diuretic, as recommended in the ACVIM guidelines for the treatment of chronic stage C MMVD, did not have a significant effect on survival time in dogs with CHF caused by MMVD. Therefore, recommending TT as standard treatment for such dogs does not seem justified.

## CONFLICT OF INTEREST DECLARATION

Authors declare no conflict of interest.

## OFF‐LABEL ANTIMICROBIAL DECLARATION

Authors declare no off‐label use of antimicrobials.

## INSTITUTIONAL ANIMAL CARE AND USE COMMITTEE (IACUC) OR OTHER APPROVAL DECLARATION

Authors declare no IACUC or other approval was needed.

## HUMAN ETHICS APPROVAL DECLARATION

Authors declare human ethics approval was not needed for this study.
